# Substance use during pregnancy and risk of postpartum depression: a systematic review and meta-analysis

**DOI:** 10.3389/fpsyt.2023.1264998

**Published:** 2023-11-09

**Authors:** Malein Pacho, Claudia Aymerich, Borja Pedruzo, Gonzalo Salazar de Pablo, Eva Sesma, Marta Bordenave, Rodrigo Dieguez, Itziar Lopez-Zorroza, Jon Herrero, Maria Laborda, Aranzazu Fernandez-Rivas, Clemente Garcia-Rizo, Miguel Angel Gonzalez-Torres, Ana Catalan

**Affiliations:** ^1^Psychiatry Department, Basurto University Hospital, Osakidetza, Basque Health Service, Bilbao, Spain; ^2^Biobizkaia Health Research Institute, Barakaldo, Spain; ^3^CIBERSAM, Centro Investigación Biomédica en Red de Salud Mental, Madrid, Spain; ^4^Early Psychosis: Interventions and Clinical-Detection (EPIC) Lab, Department of Psychosis Studies, Institute of Psychiatry, Psychology and Neuroscience, King’s College London, London, United Kingdom; ^5^Department of Child and Adolescent Psychiatry, Institute of Psychiatry and Mental Health, Hospital General Universitario Gregorio Marañón, Madrid, Spain; ^6^School of Medicine, Universidad Complutense, IiSGM, CIBERSAM, Madrid, Spain; ^7^Facultad de Medicina y Odontología, University of the Basque Country, UPV/EHU, Leioa, Spain; ^8^Barcelona Clinic Schizophrenia Unit, Hospital Clinic of Barcelona, Department of Medicine, Institut de Neurociències, Universitat de Barcelona, IDIBAPS, CIBERSAM, Barcelona, Spain; ^9^NIH Oxford Health Biomedical Research Centre, Oxford, United Kingdom; ^10^Neuroscience Department, University of Basque Country (UPV/EHU), Leioa, Spain

**Keywords:** perinatal, postpartum, postpartum depression, substance use disorder, alcohol use disorder, tobacco

## Abstract

**Introduction:**

Postpartum depression (PPD) is a prevalent mental health condition affecting women globally within the first year following childbirth. Substance use during pregnancy has been associated with an increased risk of developing PPD, but the evidence remains inconclusive. This meta-analysis aims to comprehensively assess the effects of different substances on PPD risk, exploring potential modifiers and confounding factors.

**Objectives:**

To examine the proportion of PPD among substance users during pregnancy, compared to non-users, and investigate the specific risk associated with different substances (tobacco, alcohol, and non-specified substance use/multiple substance use).

**Methods:**

A systematic literature search was conducted from inception to November 2022 using the Web of Science database (Clarivate Analytics), incorporating Web of Science Core Collection, the BIOSIS Citation Index, the KCI-Korean Journal Database, MEDLINE^®^, the Russian Science Citation Index, the SciELO Citation Index, and the Cochrane Central Register of Reviews, and Ovid/PsycINFO databases. Inclusion criteria comprised original studies with pregnant women, using validated depression scales and substance use reporting.

**Results:**

Among the 26 included studies, encompassing 514,441 women, the pooled prevalence of PPD among substance users during pregnancy was 29% (95% CI 25–33). Meta-analyzes revealed an overall odds ratio (OR) of 3.67 (95% CI 2.31–5.85, *p* < 0.01) indicating a significantly higher risk of PPD among substance users compared to non-users. Subgroup analyzes demonstrated a higher risk for women with non-specified or multiple substance use (OR 4.67, 95% CI 2.59–8.41; *p* < 0.01) and tobacco use (OR 4.01, 95% CI 2.23–7.20; *p* < 0.01). Alcohol use showed a trend toward higher risk that did not reach statistical significance (OR 1.88, 95% CI 1.00–3.55; *p* = 0.051).

**Conclusion:**

This meta-analysis provides evidence of an increased risk of PPD among pregnant substance users, particularly those using multiple substances or tobacco. However, caution is needed in interpreting the association with alcohol use due to its non-significant result.

**Systematic review registration:**

This study protocol was registered at PROSPERO (registration number: CCRD42022375500).

## 1. Introduction

### 1.1. Background

Postpartum depression (PPD) is a mental health condition affecting many women worldwide within the first year following childbirth (from 10% up to 17%) (1–3). PPD is characterized by a range of depressive symptoms that can significantly impact the mother’s well-being and potentially hinder the optimal development of the infant (4–7).

Multiple risk factors have been identified concerning the development of PPD (6, 8, 9) such as low socio-economic status, substance use, poor physical health, history of depressive disorders, multiple births or preterm births. Of particular significance is the association between PPD and substance use during pregnancy (8). Women’s risk of developing a substance use disorder is highest between 18 and 29 and remains elevated throughout their reproductive years (10, 11). According to a national survey conducted in the United States in 2013, it was estimated that up to 5% of pregnant women engage in substance use (12). However, it may be underdiagnosed due to fear of stigma and the social and legal consequences of using illicit drugs during pregnancy (13).

Substance use during pregnancy, including tobacco, alcohol, cannabis, and other substances, poses immediate risks to the health of both the mother and the developing fetus (8, 14–16) Substance use during pregnancy is strongly discouraged, and pregnant women are encouraged to seek abstinence. Additionally, pregnancy can serve as a window of opportunity in which women may be more receptive to changing behaviors to safeguard their developing child (17–19). Nonetheless, despite many women successfully achieving and maintaining abstinence during pregnancy, there is a significant tendency to relapse within the first year after childbirth, a particularly crucial period for developing a strong mother-baby bond, which is essential for healthy infant development (10, 20). Substance use has also been associated with several negative outcomes in the offspring, such as mental health problems in childhood and adolescence (21, 22), increased psychosis risk (23) and metabolic health conditions (24).

For previous reasons, addressing substance use during pregnancy and providing comprehensive support for mothers with a previous history of substance use during the postpartum period is crucial to mitigate the potential negative effects of substance use on maternal well-being and infant development.

Although several studies have examined the association between substance use during pregnancy and the development of postpartum depression (PPD) (8, 25–27), no meta-analysis has provided a comprehensive evaluation of the combined effects of different substances on the risk of PPD.

We aim to examine the proportion of postpartum depression (PPD) among substance users during pregnancy, both overall and specifically for different substances. Secondly, we assess the extent to which women with substance use during pregnancy exhibit higher PPD rates compared to those without substance use, again considering overall rates and rates specific to different substances. Lastly, we explore the influence of confounding factors, such as sample characteristics, e.g., age, marital status, or primiparity, and methodological factors, including the study risk of bias in PPD rates.

## 2. Methods

This study protocol was registered at PROSPERO (registration number: CCRD42022375500). The study was conducted in accordance with “Meta-analyzes of Observational Studies in Epidemiology” (MOOSE) checklist (28) (Supplementary Table S1) and “Preferred Reporting Items for Systematic Reviews and Meta-Analyzes” (PRISMA) (29) (Supplementary Table S2), following “EQUATOR Reporting Guidelines” (30).

### 2.1. Search strategy and selection criteria

Two independent researchers (MP and BP) conducted a systematic search of the literature up until November 30, 2022. The searches were performed using the Web of Science database (Clarivate Analytics), incorporating the Web of Science Core Collection, the BIOSIS Citation Index, the KCI-Korean Journal Database, MEDLINE®, the Russian Science Citation Index, the SciELO Citation Index, and the Cochrane Central Register of Reviews, and Ovid/PsycINFO databases.

The following keywords were used: (“substance abus*” OR “substance us*” OR addict* OR “drug abuse” OR tobacco OR alcohol* OR cannabis OR THC OR cocaine OR amphetamine* OR stimulant* OR opioid* OR “illicit drugs” OR hallucinogens) AND (pregnan* OR antenatal OR prenatal OR perinatal OR postnatal) AND (“postpartum depression”).

The inclusion criteria for the systematic review and meta-analysis were: (a) individual prospective or retrospective studies with original data reporting data of postpartum depression, defined as a depressive disorder with an onset within 6 weeks after delivery (31), (b) using a validated, structured scale to measure depressive symptoms, (c) in pregnant women of any age with any legal or illegal substance use during pregnancy (32), and (d) written in English or Spanish. Exclusion criteria were: (a) reviews, clinical cases, study protocols or qualitative studies, conferential proceedings, letters, and commentaries, (b) reporting on patients on which the onset of current depression episode precedes the current pregnancy, and (c) written in languages other than English or Spanish.

Identified articles were first screened as abstracts, and after excluding those not meeting the inclusion criteria, the full texts of the remaining articles were assessed for eligibility. In case of disagreement a senior researcher (A.C.) made the final decision. The search was completed by manually searching through the references of previously published systematic reviews and meta-analyzes on the topic.

### 2.2. Data extraction

Three researchers (CA, RD, and IL-Z) independently extracted data from all the included studies. The databases were then cross-checked by an independent researcher (MP), and discrepancies were resolved by a senior researcher (AC).

A summary of selected variables included: first author and year of publication, country, recruiting period, study type (cross-sectionals, cohorts, case–control, clinical trial), sample size, age [mean ± standard deviation (SD)] for the total sample size and each subgroup, diagnostic tool for depression, type of drug used, duration of use, frequency of use, week of pregnancy in which the drug use started, week of pregnancy in which the drug use ceases, number of events (defined as PPD diagnoses in each study group), family history of substance use, parity, previous psychiatric diagnosis both recorded as a dichotomic variable and according to the DSM or ICD criteria (1, 32), and key findings. For numeric variables, mean and SD were collected.

When multiple data points were available in one study, the latest point recorded within the first year after delivery was coded. Studies were examined for samples overlap, determined by looking at the inclusion dates and type of population and country in which the study was carried out; in case of overlapping samples, the study with the largest sample was then selected.

### 2.3. Risk of bias (quality) assessment

Risk of bias was assessed using Newcastle-Ottawa scale (33) for cohort and cross-sectional studies (Supplementary Table S3).

### 2.4. Strategy for data synthesis and statistics

A systematic synthesis of the included studies was provided. Then, we performed two separate analyzes when allowed by the data presented in the original research. First, we performed meta-analyzes using, as primary effect size, the proportion [% and standard error (SE), when available] of PPD among substance users. Second, using those articles where a comparison control group (including women without substance use during pregnancy) was included, the odds ratio (OR) with a 95% confidence interval (CI) was calculated using the number of women with PPD and sample sizes for each sample (substance users and non-users). The comparison of effect sizes in each group was calculated using the effect size (ES) formula (34). An ES greater than 1 indicates the substance-user group has a higher risk of PPD than the non-user group.

In both analyzes all the available substances were pooled for a single analysis, and subgroup meta-analyzes were subsequently conducted for each substance where data allowed for it.

Meta-regressions were performed when a minimum of 7 papers were available to study the effects of (a) mean age of the sample, (b) Newcastle-Ottawa Scale (NOS) score, (c) % of married women of the sample, and (d) % of primiparous women. Subgroup analyzes were performed to study the influence of (a) depression rating scale, and (b) used substance on the outcomes. Heterogeneity among studies was assessed using Q statistics, with the proportion of the total variability in effect size estimates evaluated using the *I*^2^ index, classifying the heterogeneity as low (*I*^2^ = 25%), medium (*I*^2^ = 50%), and high (*I*^2^ = 75%) (35). Since heterogeneity was expected to be high, the random-effect model was used. Publication bias was assessed by visually inspecting funnel plots.

All analyzes were conducted within R software, version 1.4.1106 (36). The significance level was set at *p* < 0.05, two-sided.

## 3. Results

**Figure 1:**
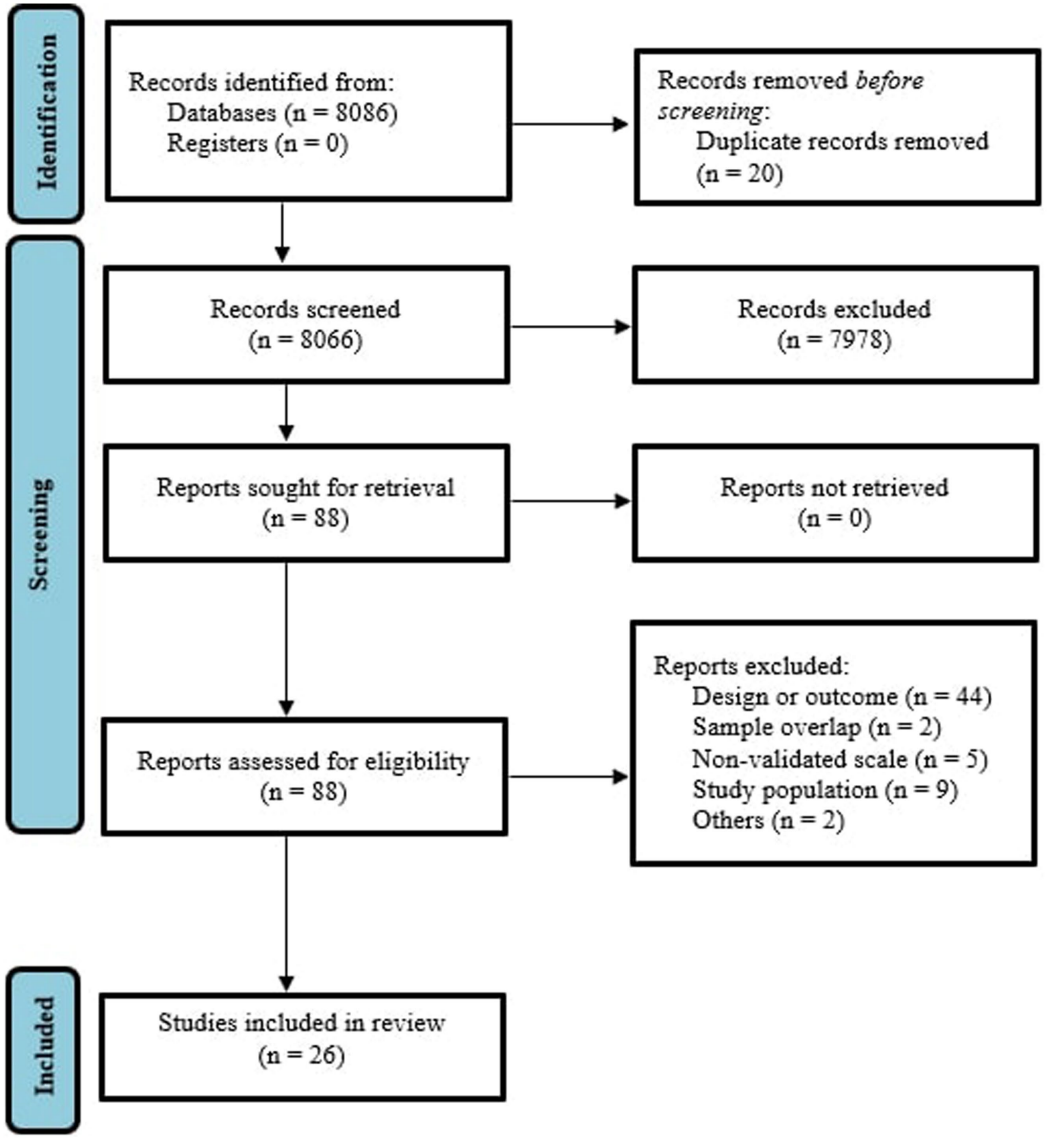
PRISMA 2009 flow diagram (25).

The literature search yielded 8,086 citations through electronic database, which were screened for eligibility; 88 articles were assessed in full text, and 62 were excluded. The final database for the systematic review and meta-analysis included 26 studies, as it can be seen in Figure 1 (29). A total sample of 45,914 women with substance use during pregnancy were included, with a mean age of 27.7 ± 3.1. 73.7% were married and 47.4% were primiparous. 61.5% of the studies used Edinburgh Postnatal Depression Scale (EDPS) (37) to rate depressive symptoms and the 38.5% of the studies used other criteria (mainly PHQ-2 scale and ICD-10 diagnostic criteria (32, 38)). 18 studies also included a control comparison group (encompassing a total of 468,527 women without substance use during pregnancy) thus allowing the calculation of an odds ratio for perinatal depression. Mean NOS score of the included studies was 6.6 ± 0.9 (Supplementary Table S4).

### 3.1. Prevalence of postpartum depression among women with substance use during pregnancy

Data were extracted for a total sample size of 36,008 women in 26 studies. 8 studies reported on women with alcohol use during pregnancy (39–45); 13 on women with tobacco use (39–49), and 10 (43.5%) on women with non-specified or multiple substance use (45, 50–56). The latest group included samples of pregnant women reporting multiple, non-specified use of legal drugs such as alcohol, tobacco and khat (50, 53, 54, 56) as well as non-specified illegal drugs, including amphetamines, cocaine and opioids (45, 51, 52, 55, 57, 58).

**Figure 2:**
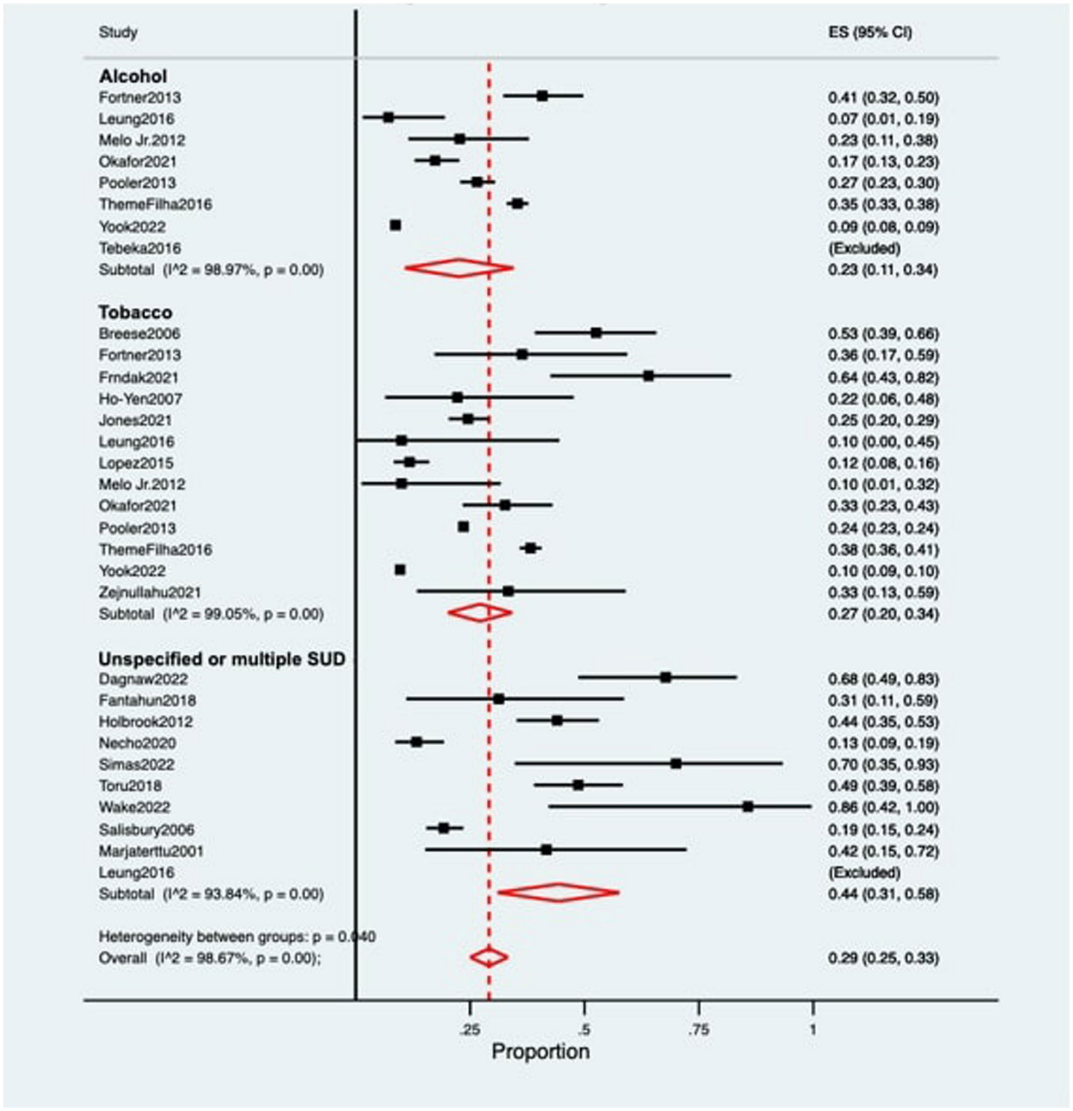
Forest plot for the prevalence of postpartum depression among women with substance use during pregnancy. ES, Effect Size; CI, Confidence Interval; SUD, Substance Use Disorder.

The pooled prevalence of postpartum depression (PPD) among women with substance use was 0.29 [95% confidence intervals (CI) 0.25–0.33; Figure 2]. When stratified by substance, women with alcohol use while pregnancy (*n* = 10,073) presented a prevalence of PPD of 0.23 (95% CI 0.11–0.34), while women using tobacco (*n* = 25,065) showed a prevalence of 0.27 (95% CI 0.20–0.34). Women with non-specified substance use or using multiple substances (besides alcohol and/or tobacco) during pregnancy (*n* = 870) showed the highest rates of PPD, at 0.44 (95% CI 0.31–0.58). Heterogeneity was significant across all of the meta-analyzed substances (*p* < 0.05 for tobacco, alcohol and non-specified or multiple substance use), as well as on the pooled sample (*p* < 0.05; Table 1).

**Table 1:** Prevalence of postpartum depression among women with substance use during pregnancy.

Substance	No. studies	Sample size	Proportion	95% CI	*p* value	z Score	I^2^ (%)
Alcohol	8	10,073	0.23	0.11–0.34	0.00*	3.72	98.97%
Tobacco	13	25,065	0.27	0.20–0.34	0.00*	7.60	99.05%
Non-specified or multiple SUD	10	870	0.44	0.31–0.58	0.00*	6.52	93.84%
Overall	23	36,008	0.29	0.25–0.33	0.00*	13.83	98.67%

CI, Confidence Interval; SUD, Substance Use Disorder. *Indicates statistical significance.

### 3.2. Odds ratio of postpartum depression among women with substance use during pregnancy compared to non-user pregnant women

**Figure 3:**
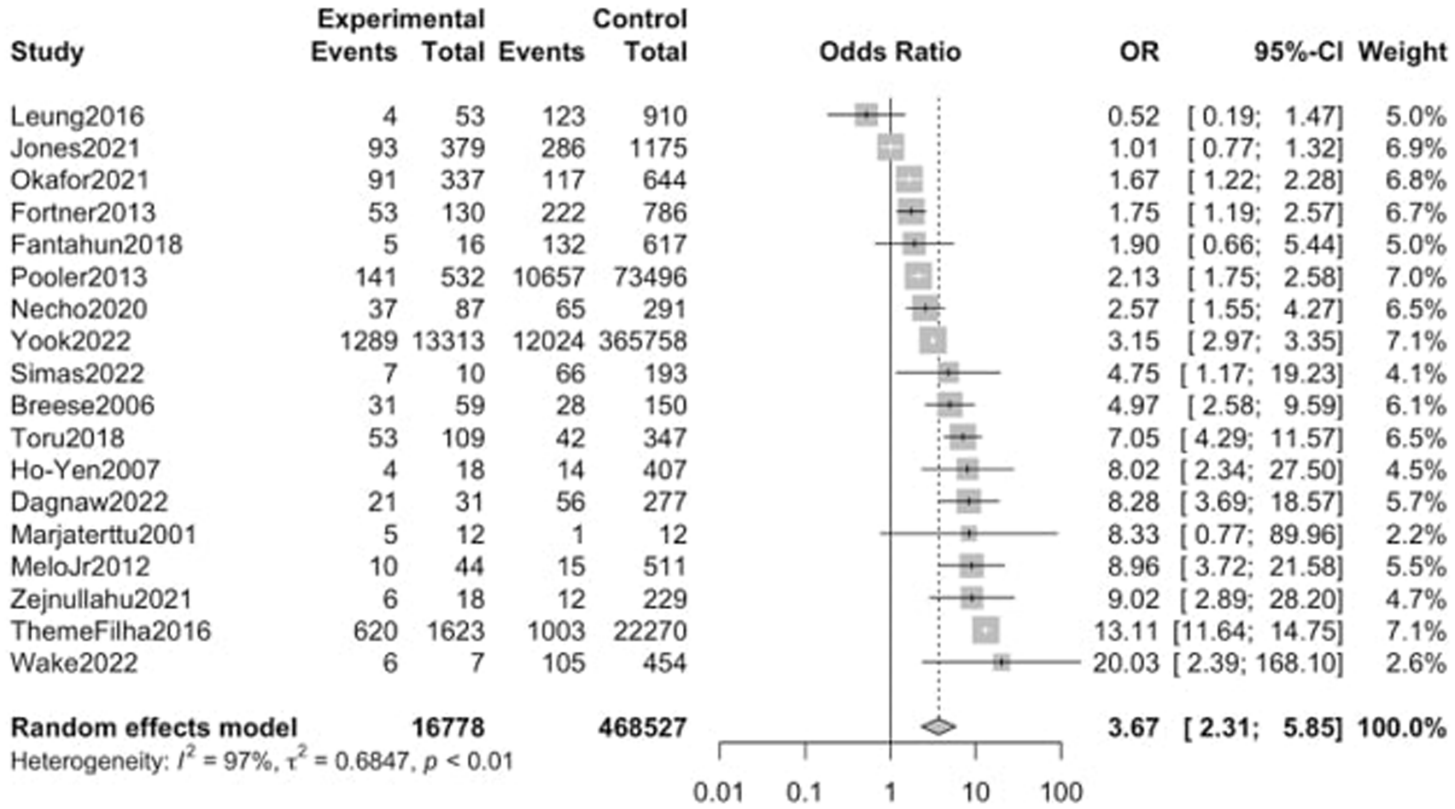
Forest plot for the odds ratio of postpartum depression among women with any substance abuse during pregnancy vs. women without. OR, Odds Ratio; CI, Confidence Interval.

**Figure 4:**
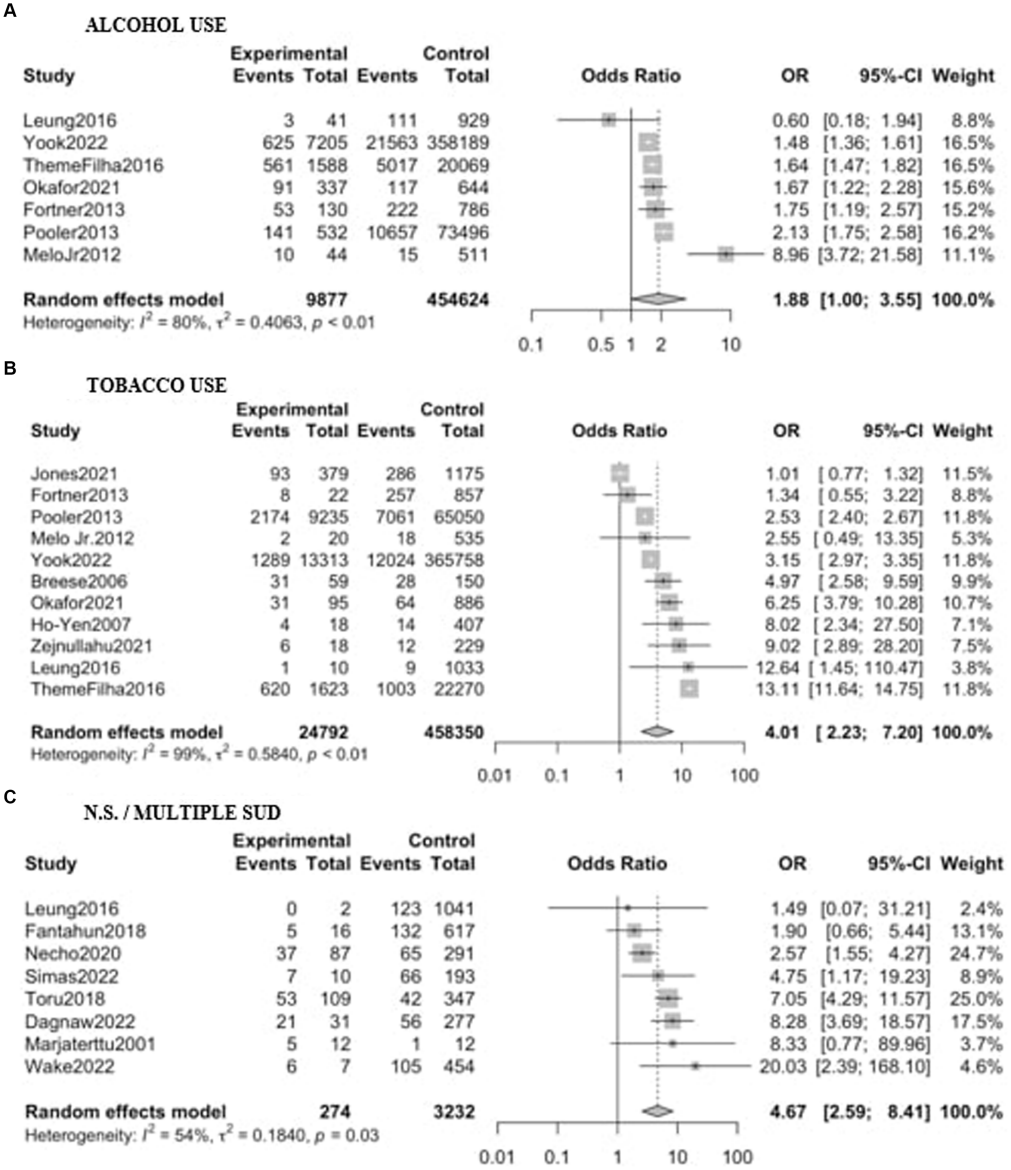
Forest plot for the odds ratio of postpartum depression among women with substance use during pregnancy vs. women without. Results are shown stratified by the substance used: **(A)** Alcohol; **(B)** Tobacco; **(C)** Non-specified/multiple substance use. OR, Odds Ratio; CI, Confidence Interval.

Eighteen studies, including a sample of 485,305 women (16,778 with substance use during pregnancy and 468,527 non-users) were included. As shown in Figure 3, PPD prevalence was higher among women with substance use during pregnancy, with an OR of 3.67 (95% CI 2.31–5.85). When analyzed by substance Figure 4, women with non-specified or multiple substance use other than alcohol and/or tobacco (*k* = 8) presented the highest risk of PPD compared to non-users, with an OR of 4.67 (95% CI 2.59–8.41, *p* < 0.01), followed by women with tobacco use (*k* = 11), who showed an OR of 4.01 (95% CI 2.23–7.20, *p* < 0.01). Finally, women with alcohol use during pregnancy (*k* = 7) did not show a statistically significant difference with those without, although a trend toward significance was detected OR of PPD of 1.88 (95% CI 0.99–3.55, *p* = 0.051). Again, heterogeneity was significant (*p* < 0.05) across all of the meta-analyzed substances, as well as on the pooled sample (Table 2).

**Table 2:** Odds ratio of postpartum depression among women with substance use during pregnancy compared to women without.

Substance	No. studies	OR	95% CI	*p* value	Test for heterogeneity		
					Q	*I*^2^ (%)	*p*
Alcohol	7	1.88	0.99–3.55	0.051	29.4	79.6	<0.01*
Tobacco	11	4.01	2.23–7.20	<0.01*	707.0	98.6	<0.01*
Non-specified or multiple SUD	8	4.67	2.59–8.41	<0.01*	15.24	54.1	0.03*
Overall	18	3.67	2.31–5.85	<0.01*	664.2	97.4	<0.01*

OR greater than 1 reflect higher prevalence of postpartum depression among women with substance use. OR Odds Ratio; CI Confidence Interval; SUD Substance Use Disorder. * Indicates statistical significance.

Meta-regressions showed no significant effect of age, NOS score, or % of primiparous women. Percentage of married women positively correlated with a greater OR of PPD (β 1.23; SE 0.58; *p* 0.04) for women with substance use (Table 3). Sensitivity analyzes showed no significant influence of the used depression rating scale on the outcome. Visual inspection of funnel plots did not suggest the presence of any publication bias for the analyzed groups (Supplementary Figures S1–S4).

**Table 3:** Meta-regressions.

	No. of Studies	β Coefficient	SE	95% CI		*Z*-Value	*P* value
Mean age	12	−0.04	0.07	−0.18	0.10	−0.51	0.61
NOS score	27	0.22	0.16	−0.10	0.53	1.35	0.18
% Married	12	1.23	0.58	0.08	2.37	2.10	0.04*
% Primiparous	14	0.00	0.01	−0.02	0.01	−0.39	0.70

## 4. Discussion

To the best of the authors’ knowledge, this is the first systematic review and meta-analysis examining both the prevalence of PPD in pregnant substance users and their odds ratio of PPD compared to non-users. The primary finding of this meta-analysis is the high prevalence of postpartum depression among substance-using pregnant women [OR 3.67, (95% CI 2.31–5.85)]. According to our analysis, a significant proportion (29%) of pregnant women who consume substances experience PPD, which is notably higher compared to other studies examining the prevalence of PPD in the general population, believed to be around 17% (3). Those reporting multiple concomitant use of legal and/or illegal substances showed the highest rate of PPD (34%), followed by women using tobacco (27%) and alcohol (23%).

Among all the potential confounding variables, only a significant effect of marital status was found, with a higher risk of PPD among samples with greater rates of married women. This finding may appear counterintuitive because the literature has reported a higher prevalence of PPD among those with less social support (59). However, several mediating factors, such as low perceived social support or marital dissatisfaction, which have been previously reported to be risk factors for postpartum depression (60, 61). This result could also potentially be attributed to the effect of domestic violence among married women, which would increase the risk of suffering PPD (62, 63). Unfortunately, we could not verify this hypothesis due to a lack of data in the articles included in our study.

### 4.1. Multiple and non-specified substance use

Women reporting the use of multiple legal and/or illegal substances during pregnancy presented the highest odds ratio for developing PPD [OR 4.67, (95% CI 2.59–8.41)]. These results align with previous findings reported in the literature, supporting the notion that substance use during pregnancy is a significant risk factor for PPD. As highlighted in the review by Pentecost (8), a substantial percentage of women with a history of substance use experience postpartum depressive symptoms, with estimates ranging between 20 and 60%. Furthermore, the study conducted by Onah et al. (64) showed that 18% of pregnant women who used alcohol and/or other drugs were currently experiencing a major depressive episode.

It is widely known that up to 1/3 of individuals with mental disorders may have comorbid substance use (65). Additionally, in women, the comorbidity between substance use and depression is higher than in men (65), partly due to the greater prevalence of affective disorders in women (66). Several theories have been proposed to explain this association. One theory suggests (53, 55) consumption of multiple substances alters brain neuroplasticity, which may contribute to the development of depressive disorders (67, 68). Another theory suggests that substance use and depression may be distinct manifestations of the same underlying neurobiological disorders (67, 68). Lastly, other studies show that may be a significant overlap between environmental factors impacting substance use and depression (67, 69) where stress may play a crucial role in this association, as it heightens the risk of both substance dependence and relapse (70) along with the occurrence of depressive episodes (55, 56, 67, 71).

### 4.2. Tobacco use

Women reporting tobacco use during pregnancy showed an OR of 4.01 (95% CI 2.23–7.20, *p* < 0.05) of PPD compared with non-users, with a total prevalence of postpartum depression of around 27%. Tobacco smoking in pregnant women had previously been linked not only to greater rates of depression (72, 73) and anxiety (74) but also to increased suicidal ideation (75).

There are several explanations for this. Tobacco use during pregnancy is linked to disturbances in the intricate neuro-hormonal balance and neurochemical pathways involved in mood regulation, including a reduction in the levels of dopamine and GABA neurotransmitters (76) and an alteration of nicotinic acetylcholine receptors involved in the hypothalamic–pituitary–adrenal (HPA) axis (77). Nicotine administration has been found to enhance the HPA axis response to stress (78, 79), a known risk factor for depression (80). The HPA axis also undergoes great changes during pregnancy (thus impacting the stress response) (81), which could help explain pregnant women’s particular vulnerability to tobacco exposure as suggested by our findings. Social factors could also contribute to the high rate of PPD among smokers. It has consistently been reported in the literature that lower socio-economic status has been associated with both higher smoking rates (82) and PPD (9), which could be a mediating factor. Also, tobacco smoking may be highly accepted in certain populations as a normative behavior because it serves as a coping mechanism for the challenges they encounter in their everyday lives (83, 84), which may represent a reporting bias. It has also been observed that exposure to second-hand tobacco smoke is associated with a higher risk of PPD, particularly in women aged 26 to 35 (75). Further research will be needed to analyze this relationship, which has been left out of the scope of this work to the lack of available data.

### 4.3. Alcohol use

Women with alcohol use during pregnancy presented a 23% rate of PPD, which is significantly higher than the PPD prevalence among the general population, reported around 17% (3). However, no significant difference was found between alcohol users and non-users in our sample, although a clear trend was found (OR 1.88; 95% CI 0.99–3.55; *p* 0.051). Our results differ from the findings of another specific meta-analysis conducted on this topic (23). That study, which presented broader inclusion criteria, reported a significant association between maternal alcohol consumption and the risk of developing PPD (27).

To address such disparities, along with a surprisingly low, non-significant, OR compared to other substances analyzed in this work such as tobacco, it is important to note several facts. First, it is essential to recognize that during pregnancy, alcohol consumption is judged more harshly than in other contexts. Therefore, many women may be reluctant to disclose their consumption during interviews, resulting in inaccurate reporting and contributing to an underestimation of alcohol use in pregnant women (85). Also, the studies included in our systematic review and meta-analysis were significantly heterogenous in their assessment of alcohol consumption across a broader range of categories, including low to moderate levels. For instance, some studies measured alcohol intake during pregnancy as a dichotomic variable (40, 43, 45), while others used specific instruments to assess severity, such as ASSIST (86) or TWEAK (42, 44, 87). Others included a threshold of intake from which alcohol use was reported (39, 41). Although not enough data was found to assess the effect of the amount of the intake on PPD prevalence or OR, this, along with the limited size of our sample, could have influenced the overall risk estimate.

Comorbidity between depression and alcohol use occurs in both directions and common but not fully understood pathophysiological processes have been postulated to explain their co-occurrence (88). For instance, it is known that they may share a common genetic susceptibility (88–90). Additionally, dysfunction in the reward and stress systems has been identified as a potential shared pathophysiology for these conditions (91).

### 4.4. Strengths and limitations

Our study offers several advantages compared to previous reviews on substance use during pregnancy (8, 10, 11, 25, 92). Firstly, we examine both the prevalence and the relative risk of postpartum depression (PPD) among pregnant substance users. Furthermore, it includes articles reporting on samples from diverse countries across six continents, which enables the analysis of different populations with distinct cultural values and varying levels of socio-economic development, enhancing the generalizability of the results and providing a more comprehensive understanding of the impact of substance use during pregnancy on the risk of PPD. Moreover, our analyzes assess the specific risk associated with different substances, including alcohol (OH), tobacco, and the combination of legal and illegal substances. By considering these categories separately, we can discern their individual contributions to the risk of PPD.

However, this meta-analysis presents also several limitations. First, a significant proportion of the included articles had a NOS score of 6 or less (38,46%, mean NOS 6.6 ± 0.9), indicating a high risk of bias. Many of the included studies were primarily focused on investigating other primary outcomes, but they included an analysis of substance use as one of the factors examined. Consequently, the available data might not fully capture all the variables relevant to our current study, therefore limiting the precision and reliability of our findings. Second, some studies had small sample sizes of pregnant women actively using substances, further impacting the statistical power of the results. Third, a high heterogenicity was found for all the analyzed variables due to the considerable variability in the samples, the scales utilized to measure PPD, and the different cut-off points employed in various studies. Fourth, our analysis was limited by the absence of available data on potential confounding variables that could influence the observed relationship. Variables such as socio-economic status (92, 93), experiences of obstetric violence (94), gender-based violence (54, 62), lack of external social support (59), obstetric factors (92–94) and pre-existing psychiatric history (54, 62, 95) have been identified in previous studies as potential confounding risk factors within the scope of our investigation. Fifth, the inclusion of the “non-specified or multiple substance use” subgroup introduces an additional layer of complexity and potential bias. While all efforts were made to avoid excluding important evidence from our analysis, this category is inherently heterogeneous, encompassing individuals with varying substance use patterns and profiles, which challenges the interpretation of the findings.

We acknowledge the complexity of research on this topic due to challenges associated with self-reporting substance use during pregnancy, including the fear of stigma or potential consequences (13). However, it is crucial to conduct more studies specifically dedicated to analyzing the relationship between substance use during pregnancy and postpartum depression, using standardized scales and measures of PPD and controlling for all said variables to provide a more comprehensive understanding of the complex interplay between substance use during pregnancy and postpartum depression. Conducting longitudinal studies would enable researchers to examine the temporal relationship, obtain valuable insights into the causal pathways involved and help identify critical periods for targeted intervention.

## 5. Conclusion

In conclusion, this systematic review and meta-analysis demonstrate an alarming prevalence of postpartum depression among pregnant substance users, extending beyond illegal substances to legal ones. It is particularly concerning to note the high prevalence of PPD among women who smoke tobacco, given that tobacco is a legal and socially accepted substance.

The findings underscore the urgent need for intensified monitoring, early intervention, and tailored support for pregnant women who consume legal or illegal substances. Additionally, there is a clear call for future prospective and high-quality studies to explore further the complex relationships between substance use, mediating factors, and PPD. By addressing these gaps in knowledge, healthcare professionals and policymakers should develop evidence-based strategies to mitigate the risks associated with substance use during pregnancy while improving not only maternal mental health but also considering the offspring’s mental and physical conditions.

## Author contributions

MP: Conceptualization, Investigation, Methodology, Project administration, Writing – original draft, Writing – review & editing. CA: Conceptualization, Data curation, Writing – original draft, Writing – review & editing, Formal analysis. BP: Data curation, Investigation, Methodology, Writing – review & editing. GS: Conceptualization, Methodology, Writing – review & editing. ES: Conceptualization, Writing – review & editing. MB: Conceptualization, Investigation, Writing – review & editing. RD: Conceptualization, Investigation, Writing – review & editing. IL-Z: Conceptualization, Investigation, Writing – review & editing. JH: Conceptualization, Investigation, Writing – review & editing. ML: Conceptualization, Investigation, Writing – review & editing. AF-R: Conceptualization, Writing – review & editing. CG-R: Conceptualization, Writing – review & editing. MG-T: Supervision, Validation, Writing – review & editing. AC: Conceptualization, Formal analysis, Software, Supervision, Validation, Writing – review & editing.
