# Supplementary material for: Substance use during pregnancy and risk of postpartum depression: a systematic review and meta-analysis
Source: Front Psychiatry. 2023 Nov 9;14:1264998. doi: 10.3389/fpsyt.2023.1264998 (PMC10666188; doi:10.3389/fpsyt.2023.1264998)
Supplement: Supplementary file 1 [file Data_Sheet_1.docx]

Supplementary Material

Substance use during pregnancy and risk of postpartum depression: a systematic review and meta-analysis

Malein Pacho*, Claudia Aymerich*, Borja Pedruzo, Gonzalo Salazar de Pablo, Eva Sesma, Marta Bordenave, Rodrigo Dieguez, Itziar Lopez-Zorroza, Jon Herrero, Maria Laborda, Aranzazu Fernandez-Rivas, Clemente Garcia-Rizo , Miguel Angel Gonzalez-Torres†, Ana Catalan†

*** Correspondence:** Corresponding Author: Malein Pacho; [malein.pachogarcia@osakidetza.eus](mailto:malein.pachogarcia@osakidetza.eus)

# Supplementary Figures
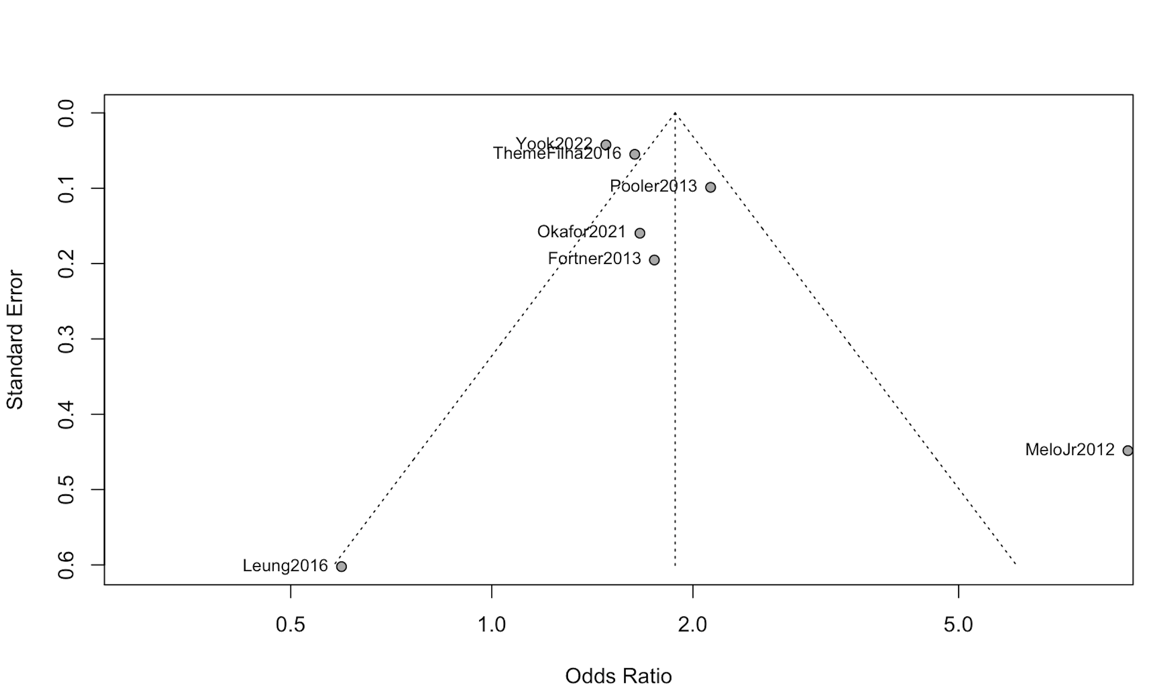


# Supplementary Figure 1 . Funnel plot analysing publication bias in the articles included for the analysis of alcohol use in pregnancy and risk of postpartum depression.


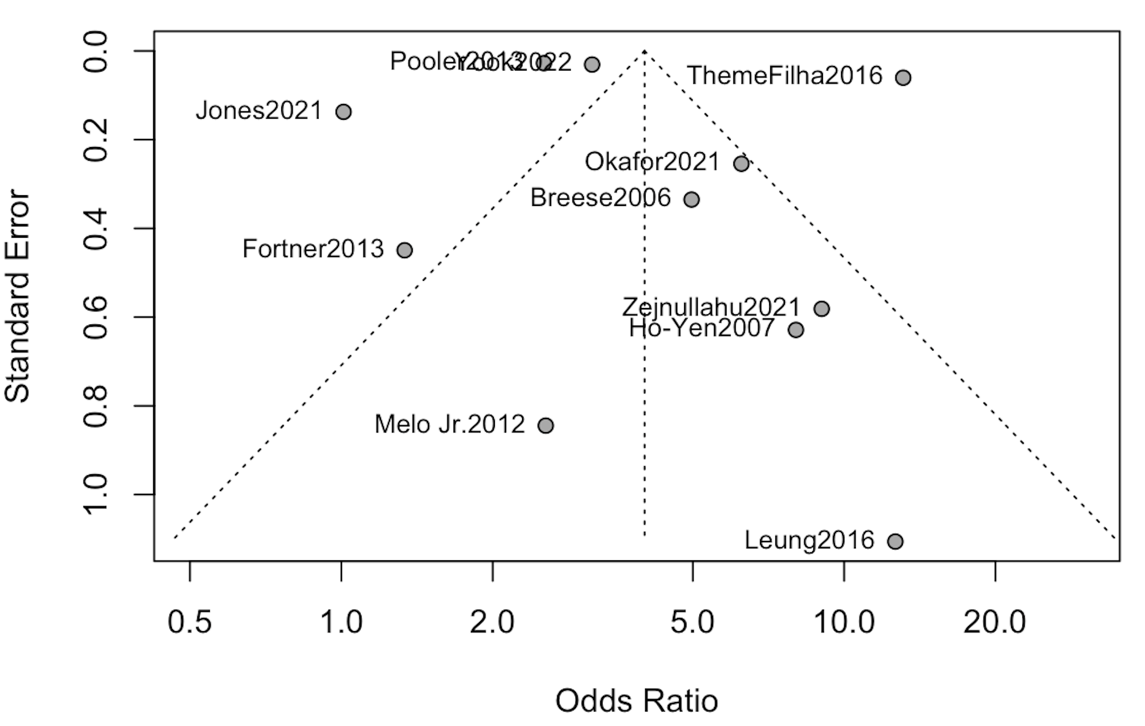


Supplementary Figure 2. Funnel plot analysing publication bias in the articles included for the analysis of tobacco use in pregnancy and risk of postpartum depression.


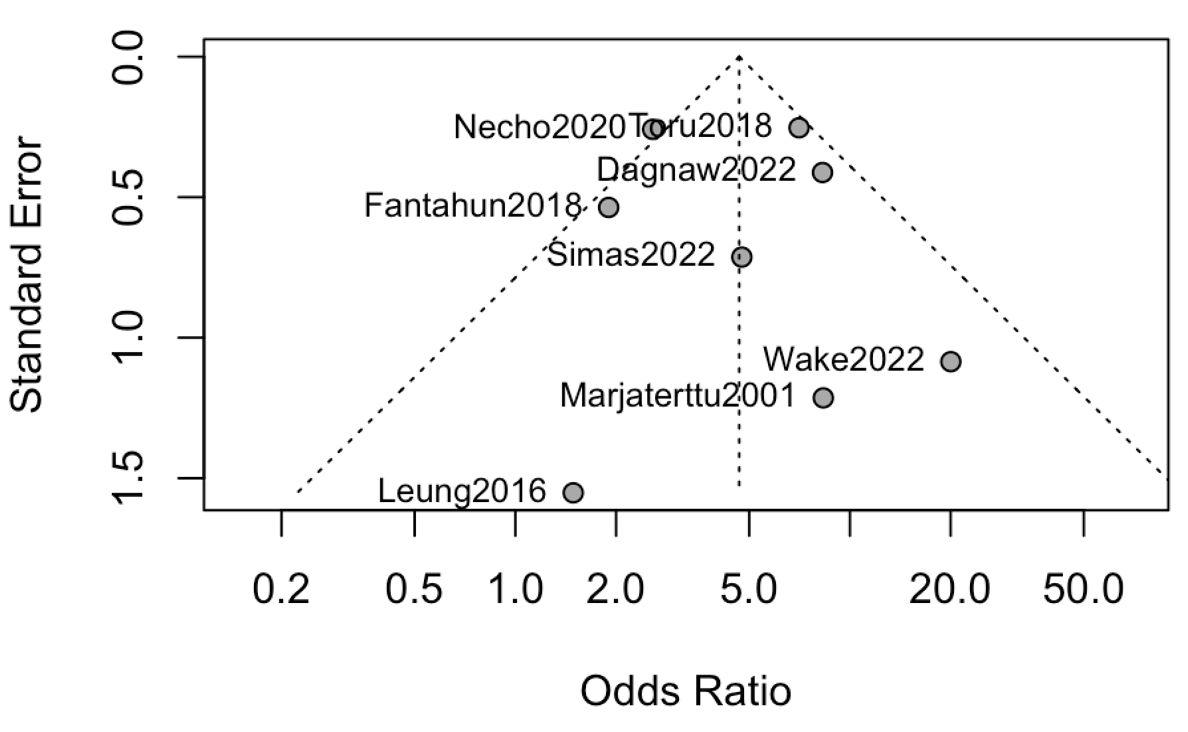


Supplementary Figure 3. Funnel plot analysing publication bias in the articles included for the analysis of N.S. / Multiple SUD in pregnancy and risk of postpartum depression.


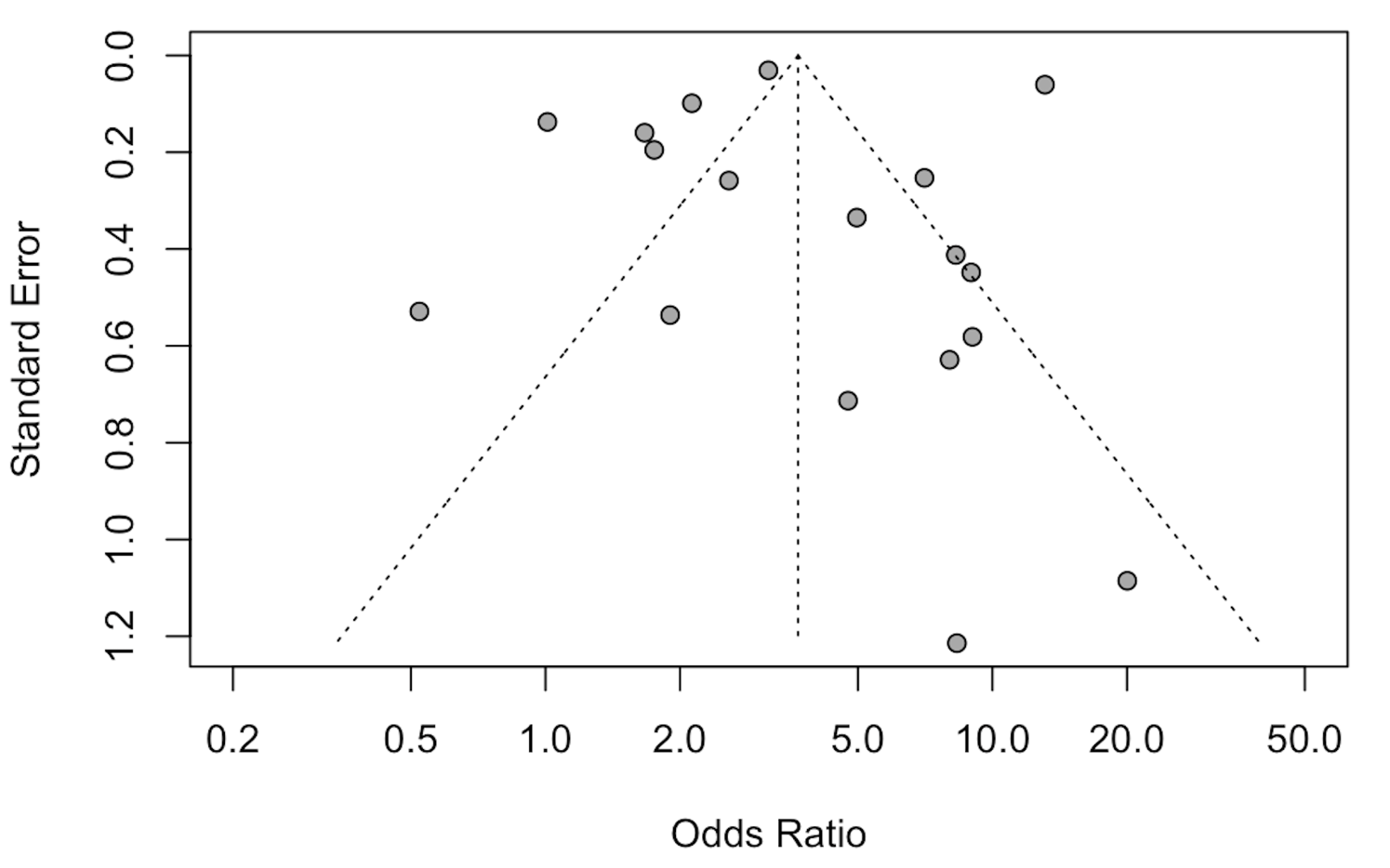


Supplementary Figure 4. Funnel plot analysing the publication bias of all the articles included in the study.

# Tables

**Supplementary Table 1: Prisma statement and checklist**

| **Section/Topic** | **Item #** | **Checklist item** | **YES/NO** |
| --- | --- | --- | --- |
| **TITLE** | | | |
| Title | 1 | Identify the report as a systematic review. | YES |
| **ABSTRACT** |  |  |  |
| Abstract | 2 | Provide a structured summary including, as applicable: background; objectives; data sources; study eligibility criteria, participants, and interventions; study appraisal and synthesis methods; results; limitations; conclusions and implications of key findings; systematic review registration number. | YES |
| **INTRODUCTION** | | | |
| Rationale | 3 | Describe the rationale for the review in the context of existing knowledge. | YES |
| Objectives | 4 | Provide an explicit statement of the objective(s) or question(s) the review addresses. | YES |
| **METHODS** | | | |
| Eligibility criteria | 5 | Specify the inclusion and exclusion criteria for the review and how studies were grouped for the syntheses. | YES |
| Information sources | 6 | Specify all databases, registers, websites, organisations, reference lists and other sources searched or consulted to identify studies. Specify the date when each source was last searched or consulted. | YES |
| Search strategy | 7 | Present the full search strategies for all databases, registers and websites, including any filters and limits used. | YES |
| Selection process | 8 | Specify the methods used to decide whether a study met the inclusion criteria of the review, including how many reviewers screened each record and each report retrieved, whether they worked independently, and if applicable, details of automation tools used in the process. | YES |
| Data collection process | 9 | Specify the methods used to collect data from reports, including how many reviewers collected data from each report, whether they worked independently, any processes for obtaining or confirming data from study investigators, and if applicable, details of automation tools used in the process. | YES |
| Data items | 10 | List and define all outcomes for which data were sought and if any assumptions were made about any missing or unclear information. | YES |
| Study risk of bias assessment | 11 | Specify the methods used to assess risk of bias in the included studies, including details of the tool(s) used, how many reviewers assessed each study and whether they worked independently, and if applicable, details of automation tools used in the process. | YES |
| Effect measures | 12 | Specify for each outcome the effect measure(s) (e.g. risk ratio, mean difference) used in the synthesis or presentation of results. | YES |
| Synthesis methods | 13 | Describe the processes used to decide which studies were eligible for each synthesis. Describe any methods required to prepare the data for presentation or synthesis, such as handling of missing summary statistics, or data conversions. Describe any methods used to tabulate or visually display results of individual studies and syntheses. Describe any methods used to synthesize results and provide a rationale for the choice(s). Describe the model(s), method(s) to identify the presence and extent of statistical heterogeneity, and software package(s) used, any methods used to explore possible causes of heterogeneity among study results (e.g. subgroup analysis, meta-regression) and any sensitivity analyses conducted to assess robustness of the synthesized results. | YES |
| Reporting bias assessment | 14 | Describe any methods used to assess risk of bias due to missing results in a synthesis (arising from reporting biases). | YES |
| Certainty assessment | 15 | Describe any methods used to assess certainty (or confidence) in the body of evidence for an outcome. | YES |
| **RESULTS** | | | |
| Study selection | 16 | Describe the results of the search and selection process, from the number of records identified in the search to the number of studies included in the review, ideally using a flow diagram. Cite studies that might appear to meet the inclusion criteria, but which were excluded, and explain why they were excluded. | YES |
| Study characteristics | 17 | Cite each included study and present its characteristics. | Supl Table |
| Risk of bias in studies | 18 | Present assessments of risk of bias for each included study. | Supl Table |
| Results of individual studies | 19 | For all outcomes, present, for each study: (a) summary statistics for each group (where appropriate) and (b) an effect estimate and its precision (e.g. confidence/credible interval), ideally using structured tables or plots. | Supl Table |
| Results of syntheses | 20 | For each synthesis, briefly summarise the characteristics and risk of bias among contributing studies. Present results of all statistical syntheses conducted. If meta-analysis was done, present for each the summary estimate and its precision (e.g. confidence/credible interval) and measures of statistical heterogeneity. If comparing groups, describe the direction of the effect. Present results of all investigations of possible causes of heterogeneity among study results and all sensitivity analyses conducted to assess the robustness of the synthesized results. | Supl Table |
| Reporting biases | 21 | Present assessments of risk of bias due to missing results (arising from reporting biases) for each synthesis assessed. | Supl Table |
| Certainty of evidence | 22 | Present assessments of certainty (or confidence) in the body of evidence for each outcome assessed. | YES |
| **DISCUSSION** | | | |
| Discussion | 23 | Provide a general interpretation of the results in the context of other evidence. Discuss any limitations of the evidence included in the review, any limitations of the review processes used and the implications of the results for practice, policy, and future research. | YES |
| **OTHER INFORMATION** | | | |
| Registration and protocol | 24 | Provide registration information for the review, including register name and registration number, or state that the review was not registered. Indicate where the review protocol can be accessed, or state that a protocol was not prepared. Describe and explain any amendments to information provided at registration or in the protocol. | YES |
| Support | 25 | Describe sources of financial or non-financial support for the review, and the role of the funders or sponsors in the review. | YES |
| Competing interests | 26 | Declare any competing interests of review authors. | YES |
| Availability of data, code and other materials | 27 | Report which of the following are publicly available and where they can be found: template data collection forms; data extracted from included studies; data used for all analyses; analytic code; any other materials used in the review. | N.A |

**Supplementary Table 2: MOOSE Statement - Reporting Checklist for Authors, Editors, and Reviewers of Meta-analyses of Observational Studies**

| **Reporting Criteria** | **Reported (Yes/No)** |
| --- | --- |
| **Reporting of Background** |  |
| Problem definition | Yes |
| Hypothesis statement | Yes |
| Description of Study Outcome(s) | Yes |
| Type of exposure or intervention used | Yes |
| Type of study design used | Yes |
| Study population | Yes |
| **Reporting of Search Strategy** |  |
| Qualifications of searchers (eg, librarians and investigators) | Yes |
| Search strategy, including time period included in the synthesis and keywords | Yes |
| Effort to include all available studies, including contact with authors | Yes |
| Databases and registries searched | Yes |
| Search software used, name and version, including special features used (eg, explosion) | Yes |
| Use of hand searching (eg, reference lists of obtained articles) | NO |
| List of citations located and those excluded, including justification | NO |
| Method for addressing articles published in languages other than English | YES |
| Method of handling abstracts and unpublished studies | NO |
| Description of any contact with authors | NO |
| **Reporting of Methods** |  |
| Description of relevance or appropriateness of studies assembled for assessing the hypothesis to be tested | Yes |
| Rationale for the selection and coding of data (eg, sound clinical principles or convenience) | Yes |
| Documentation of how data were classified and coded (eg, multiple raters, blinding, and interrater reliability) | Yes |
| Assessment of confounding (eg, comparability of cases and controls in studies where appropriate | Yes |
| Assessment of study quality, including blinding of quality assessors; stratification or regression on possible predictors of study results YES 5 | Yes |
| Assessment of heterogeneity | Yes |
| Description of statistical methods (eg, complete description of fixed or random effects models, justification of whether  the chosen models account for predictors of study results, dose-response models, or cumulative meta-analysis) in sufficient detail to be replicated | Yes |
| Provision of appropriate tables and graphics | Yes |
| **Reporting of Results** |  |
| Table giving descriptive information for each study included | Yes |
| Results of sensitivity testing (eg, subgroup analysis) | Yes |
| Indication of statistical uncertainty of findings | Yes |
| **Reporting of Discussion** |  |
| Quantitative assessment of bias (eg, publication bias) | Yes |
| Justification for exclusion (eg, exclusion of non–English-language citations) | Yes |
| Assessment of quality of included studies | Yes |
| **Reporting of Conclusions** |  |
| Consideration of alternative explanations for observed results | Yes |
| Generalization of the conclusions (ie, appropriate for the data presented and within the domain of the literature review) | Yes |
| Guidelines for future research | Yes |
| Disclosure of funding source | Yes |

**Supplementary Table 3: Quality assessment: Newcastle-Ottawa Scale (NOS) for Cohort Studies**

Quality of the included studies was assessed using the Newcastle-Ottawa Scale (NOS) for Cohort Studies due to the heterogeneity expected in the included studies. The following assessment scores were used:

| **Criteria** | **Maximum Score** |
| --- | --- |
| Representativeness of exposed cohort | ★ |
| Selection of the non-exposed cohort | ★ |
| Ascertainment of exposure | ★ |
| Demonstration that outcome of interest was not present at start of study | ★ |
| Comparability of cohorts on the basis of the design or analysis controlled for confounders | ★ ★ |
| Assessment of outcome | ★ |
| Was follow-up long enough for outcomes to occur | ★ |
| Adequacy of follow-up of cohorts | ★ |

**Supplementary Table 4**. Table with extraction details for each substance. **N.** Number of patients included.. **PPD Scale**: Postpartum depression scale.

| **ALCOHOL** | | | | | | | | | | | | | | | | | | | | | | | | | | | | | | | | | |
| --- | --- | --- | --- | --- | --- | --- | --- | --- | --- | --- | --- | --- | --- | --- | --- | --- | --- | --- | --- | --- | --- | --- | --- | --- | --- | --- | --- | --- | --- | --- | --- | --- | --- |
| **Author_Year** | **Year** | | **N total sample** | | **Mean age total sample** | **SD mean age total sample** | | **N Pregnancy substance use** | | **N Non consumer pregnants** | | **PPD Scale** | | | | **CUT OFF PPD** | | **OR PSU** | | | **CI 95% PPD PSU** | | | **% PPD PSU** | | **%PPD C** | | | **% Married** | | **% Primiparity** | | **NOS** |
| Yook2022 | 2022 | | 392394 | | 30,18 | 3,73 | | 7205 | | 358189 | | ICD-10 | | | | N.A | | 1,48 | | | 1.36-1.61 | | | 8,67% | | 6,02% | | | N.A. | | N.A. | | 8 |
| Fortner2013 | 2011 | | 921 | | N.A. | N.A. | | 130 | | 786 | | EPDS | | | | 12 | | 1.75 | | | 1.19-2.57 | | | 40,80% | | 28,24% | | | 52,30% | | 19,40% | | 7 |
| Pooler2013 | 2013 | | 75234 | | N.A. | N.A. | | 532 | | 73496 | | PHQ-2 | | | | N.A | | 2.13 | | | 1.75-2.58 | | | 26,50% | | 14,50% | | | N.A. | | 41,50% | | 7 |
| ThemeFilha2016 | 2016 | | 23806 | | N.A. | N.A. | | 1588 | | 20069 | | EPDS | | | | 13 | | 1.64 | | | 1.47-182 | | | 35,30% | | 25,00% | | | 81,34% | | 46,91% | | 6 |
| Melo Jr.2012 | 2012 | | 555 | | N.A. | N.A. | | 44 | | 511 | | EPDS | | | | 13 | | 8.96 | | | 3.72-21.58 | | | 21,70% | | 3,00% | | | N.A. | | 50,06% | | 6 |
| Okafor2021 | 2021 | | 981 | | 27 | 6 | | 248 | | 733 | | EPDS | | | | 13 | | 1.67 | | | 1.22-2.28 | | | 17,30% | | 22,00% | | | N.A. | | N.A. | | 5 |
| Leung2016 | 2016 | | 1043 | | 31 | 4 | | 42 | | 1001 | | EPDS | | | | 10 | | 0.60 | | | 0.18-1.94 | | | 7,30% | | 11,80% | | | N.A. | | N.A. | | 6 |
| **TOBACCO** | | | | | | | | | | | | | | | | | | | | | | | | | | | | | | | | | |
| **Author_Year** | **Year** | | **N total sample** | | **Mean age total sample** | **SD mean age total sample** | | **N Pregnancy substance use** | | **N Non consumer pregnants** | | **PPD Scale** | | **CUT OFF PPD** | | | **OR PSU** | | | **CI 95% PPD PSU** | | **% PPD PSU** | | | **%PPD C** | | | **% Married** | | | **% Primiparity** | | **NOS** |
| Yook2022 | 2022 | | 392394 | | 30,18 | 3,73 | | 13313 | | 365758 | | ICD-10 | | N.A | | | 3.15 | | | 2.97-3.35 | | 9,68% | | | 6,02% | | | N.A | | | N.A | | 8 |
| Zejnullahu2021 | 2021 | | 247 | | 28,7 | 5,5 | | 18 | | 229 | | EPDS | | 12 | | | 9.02 | | | 2.89-28.80 | | 33,30% | | | 20,08% | | | N.A | | | 69,67% | | 6 |
| Fortner2013 | 2011 | | 921 | | N.A | N.A | | 22 | | 899 | | EPDS | | 12 | | | 1.34 | | | 0.55-3.22 | | 36,40% | | | 29,90% | | | 52,30% | | | 19,40% | | 7 |
| Pooler2013 | 2013 | | 75234 | | N.A | N.A | | 9235 | | 65050 | | PHQ-2 | | N.A | | | 2.53 | | | 2.40-6.67 | | 23,54% | | | 13,27% | | | N.A | | | 41.50% | | 7 |
| ThemeFilha2016 | 2016 | | 23806 | | N.A | N.A | | 1623 | | 22270 | | EPDS | | 13 | | | 13.11 | | | 11.64-14.75 | | 38,20% | | | 25,60% | | | 81,34% | | | 46.91% | | 6 |
| Ho-Yen2007 | 2007 | | 426 | | 24,5 | N.A | | 18 | | 407 | | EPDS | | 12 | | | 8.02 | | | 2.34-27.50 | | 22,22% | | | 4,18% | | | 100% | | | N.A | | 6 |
| Jones2021 | 2020 | | 1554 | | 22,44 | 4,67 | | 379 | | 1175 | | EPDS | | 10 | | | 1.01 | | | 0.77-1.32 | | 24,53% | | | 19,15% | | | 10% | | | N.A | | 6 |
| Melo Jr.2012 | 2012 | | 555 | | N.A | N.A | | 20 | | 535 | | EPDS | | 13 | | | 2.55 | | | 0.49-13.35 | | 8,30% | | | 6,30% | | | N.A | | | 50,06% | | 6 |
| Okafor2021 | 2021 | | 981 | | 27 | 6 | | 95 | | 886 | | EPDS | | 13 | | | 6.25 | | | 3.79-10.28 | | 32,80% | | | 22,00% | | | N.A | | | N.A | | 5 |
| Breese2006 | 2006 | | 209 | | N.A | N.A | | 59 | | 150 | | EPDS | | 13 | | | 4.97 | | | 2.58-9.59 | | 53,00% | | | 33,00% | | | N.A | | | N.A | | 6 |
| Leung2016 | 2016 | | 1043 | | 31 | 4 | | 10 | | 1033 | | EPDS | | 10 | | | 12.64 | | | 1.45-110.47 | | 10,00% | | | 11,80% | | | N.A | | | N.A | | 6 |
| **NON SPECIFIED SUBSTANCE USE** | | | | | | | | | | | | | | | | | | | | | | | | | | | | | | | | | |
| **Author_Year** | | **Year** | | **N total sample** | **Mean age total sample** | **SD mean age total sample** | **N Pregnancy substance use (substance)** | | **N Non consumer pregnants** | | **PPD Scale** | | **CUT OFF PPD** | | **OR PSU** | | | | **CI 95% PPD PSU** | | | | **% PPD PSU** | | | | **%PPD C** | | | **% Married** | | **% Primiparity** | **NOS** |
| Toru2018 | | 2018 | | 456 | N.A | N.A | 109 (Alcohol, khat, tobacco, and/or other substances) | | 347 | | PHQ-9 | | 10 | | 7.05 | | | | 4.29-11.57 | | | | 49,00% | | | | 12,00% | | | 97,10% | | N.A | 6 |
| Wake2022 | | 2022 | | 461 | N.A | N.A | 7 (Not specified) | | 454 | | EPDS | | 13 | | 20.03 | | | | 2.39-168.10 | | | | 85,70% | | | | 23,03% | | | 90,50% | | 27,10% | 7 |
| Marjaterttu2001 | | 2001 | | 24 | 26,35 | 4,63 | 12 (Abuse substances or mixed abuse substances and alcohol) | | 12 | | EPDS | | 12 | | 8.33 | | | | 0.77-89.96 | | | | 42,00% | | | | 8,00% | | | N.A | | N.A | 7 |
| Fantahun2018 | | 2018 | | 633 | N.A | N.A | 16 (Alcohol, khat, tobacco, and/or other substances) | | 617 | | EPDS | | 13 | | 1.90 | | | | 0.66-5.44 | | | | 34,10% | | | | 21,40% | | | 85,10% | | 36,90% | 6 |
| Simas2022 | | 2022 | | 203 | N.A | N.A | 10 (Not specified) | | 193 | | EPDS | | 10 | | 4.75 | | | | 1.17-19.23 | | | | 71,00% | | | | 34,00% | | | 20,00% | | 47,00% | 8 |
| Necho2020 | | 2020 | | 378 | 29,85 | 6,39 | 173 (Alcohol, khat, tobacco, and/or other substances) | | 204 | | EPDS | | 13 | | 2.57 | | | | 1.55-4.27 | | | | 13.22% | | | | N.A | | | 74,90% | | 32,80% | 8 |
| Leung2016 | | 2016 | | 1043 | 31 | 4 | 2 (Abuse substances other than alcohol and tobacco) | | 1041 | | EPDS | | 10 | | 1.49 | | | | 0.07-31.21 | | | | 0,00% | | | | 11,80% | | | N.A | | N.A | 6 |
| Dagnaw2022 | | 2022 | | 308 | N.A | N.A | 31 (Alcohol, khat, tobacco, and/or other substances) | | 277 | | EPDS | | 10 | | 8.28 | | | | 3.69-18.57 | | | | 67,70% | | | | 20,20% | | | N.A | | N.A | 8 |
